# Editorial: Constructive approach to spatial cognition in intelligent robotics

**DOI:** 10.3389/fnbot.2022.1077891

**Published:** 2022-11-15

**Authors:** Akira Taniguchi, Michael Spranger, Hiroshi Yamakawa, Tetsunari Inamura

**Affiliations:** ^1^College of Information Science and Engineering, Ritsumeikan University, Kyoto, Japan; ^2^Sony Computer Science Laboratories, Tokyo, Japan; ^3^The Whole Brain Architecture Initiative, Tokyo, Japan; ^4^School of Engineering, The University of Tokyo, Tokyo, Japan; ^5^Center for Biosystems Dynamics Research, Institute of Physical and Chemical Research (RIKEN), Osaka, Japan; ^6^Principles of Informatics Research Division, National Institute of Informatics, Tokyo, Japan; ^7^Department of Informatics, The Graduate University for Advanced Studies, SOKENDAI, Tokyo, Japan

**Keywords:** simultaneous localization and mapping, spatial reasoning, place recognition and categorization, navigation and path planning, spatial language understanding

## 1. Introduction

For agents operating in the real world, spatial reasoning and understanding of the spatial properties of the environment are important abilities for executing tasks related to spatial movement (Kostavelis and Gasteratos, 2015; Garg et al., 2020). Agents can perform various tasks by acquiring and utilizing the semantic and linguistic knowledge related to place and object locations. Current research on spatial reasoning and semantic understanding in robots is important for realizing self-localization with uncertainty in the real world and planning with human–robot interactions. It is also closely related to a constructive approach to brain-inspired AI related to spatial cognition represented by the hippocampal formation (Tolman, 1948; O'keefe and Nadel, 1978).

In this Research Topic, we address the interdisciplinary fusion of knowledge of artificial intelligence, robotics, cognitive development, and neuroscience in spatial cognition and spatial reasoning. For example, in the fusion area of natural language processing and computer vision, research on vision-and-language navigation (VLN) has recently been implemented (Anderson et al., 2018; Chen et al., 2021).

However, few studies have applied VLN technology to real-world robots. In the future, a VLN that operates in a real environment will be a necessity. Additionally, in robotics AI, referring to the cognitive and neuroscientific findings of concept formation related to place and spatial language acquisition would be useful. To achieve this, a constructive approach using robots operating in the real world would be effective (Asada et al., 2009; Cangelosi and Schlesinger, 2015; Taniguchi et al., 2019).

This Research Topic was widely assembled from fundamental to applied research, which is related to spatial reasoning using robots and semantic understanding including language interaction and in the fusion area of artificial intelligence, such as machine learning, robotics, and computational neuroscience. These papers contribute to advancing the field on the technical side, for example, semantic mapping, place recognition, and navigation for performing tasks including spatial movement. Additionally, some studies contribute to computational models related to spatial reasoning, such as referring to hippocampal formation and spatial cognitive capabilities. Furthermore, the focus is on contributions to cutting-edge machine learning for use in the above aspects.

## 2. About the Research Topic

We are pleased to present five research articles related to spatial relation learning, spatial concept formation, bio-inspired models, localization, and navigation. In this section, we briefly introduce each paper.

Autonomous mobile robots and self-driving vehicles require accurate and reliable self-localization to handle dynamic environments. Colomer et al. address the problem of visual location awareness using a neuro-cybernetic approach. The proposed method is a biologically-inspired neural network called the log-polar max-Pi (LPMP) model. In particular, the visual–spatial processing associated with the hippocampus and entorhinal cortex is referenced. A mechanism is constructed to integrate information from two parallel pathways, the “what” and “where” pathways of the visual cortex. The localization performance is evaluated in a road environment, demonstrating its usefulness compared to conventional methods.

Grid cells in the medial entorhinal cortex of the mammalian brain are essential for the path integration and representation of the external world (Hafting et al., 2005; McNaughton et al., 2006). However, it has been argued that few models explain the formation of various grid-field structures in recent relevant studies. To fill this gap, Gong and Yu propose an interpretative plane-dependent model of 3D grid cells to represent both 2D and 3D spaces. The proposed method comprises a spatial transformation using 6-DOF motion and a recurrent neural network (RNN) for 3D grid cells. In simulation experiments, a representation similar to that of hexagonal grid cells is reported to be obtained. The results validate the hypothesis that “grid fields gradually lose global but not local order as the refresh interval decreases.”

For agents to perceive and act in a physical space, learning relevant concepts about space and its environment (objects, colors, shapes, etc.) is essential. Lee et al. propose three approaches to enable cognitive agents to learn spatial relations. The proposed approach integrates (i) the learning of language–spatial relations through embodied experience, (ii) the learning of directional relations using large-scale image data, and (iii) the inference of spatial relations using a knowledge base. For learning, an upper-body humanoid robot and a neural network-based model are used. Partial experiments for each component of the proposed approach demonstrate its applicability. The authors present the concept of an integrated architecture, which still needs to be implemented and validated.

Humans are assumed to recognize continuous high-dimensional observations by segmenting and classifying them into significant segments. Nagano et al. propose HcVGH, a method for learning spatio-temporal categories by segmenting first-person-view videos captured by a mobile robot. HcVGH combines a convolutional variational auto-encoder (cVAE) with the authors' previous model, a hierarchical Dirichlet process-variational autoencoder-Gaussian process-hidden semi-Markov model (HVGH) (Nagano et al., 2019), based on a probabilistic generative model. HVGH is an unsupervised segmentation method for high-dimensional time series. The experimental results show that the proposed method is capable of classifying and segmenting sequences of robot perspective videos with high accuracy in a simulation environment. The transition probabilities estimated by HcVGH can be used for global path planning, potentially enabling the planning of different paths for different purposes.

Sentences containing spatial instructions from the user to the robot are not only based on a coordinate system that is absolute concerning the environment, such as “kitchen.” Some instructions relate to relative positions, such as “next to the chair” or “in front of the TV.” Relative spatial concepts are based on a coordinate system relative to an object or agent. In a previous study on spatial concept formation (Taniguchi et al., 2017), the main focus was only on absolute concepts. In contrast, the method proposed by Sagara et al. enables the robot to simultaneously estimate the coordinate system and spatial concepts (absolute and relative). The relative and absolute spatial concept acquisition method (RASCAM) is based on the model of the authors' previous work ReSCAM+O (Sagara et al., 2021) and the model of absolute spatial concept formation (Taniguchi et al., 2017), which is composed of a probabilistic generative model. Experiments in a simulated home environment show that the proposed approach can learn relative and absolute spatial concepts while accurately selecting the coordinate system. This approach will help the service robot flexibly understand new environments through human interaction.

We hope the above articles will interest the readers in the recent efforts in spatial cognition in robots and highlight the importance of this research field.

## 3. Next step

Many challenges remain in further developing spatial cognition and spatial semantic understanding using robots.

The first problem is that models are still often tested by simulators. To achieve intelligent models that perform robustly over the long term, they should be are tested in dynamic real-world environments with various observational noises and uncertainties. In particular, real-world applications of VLNs will rapidly advance.

The second issue is spatial linguistic semantic understanding. As discussed in a paper on our topic, learning the relationship between spatial concepts and language is an interesting field for further development. Moreover, applying large-scale language models to real-world environments is an issue to be addressed in the future.

The third challenge is the integration of advanced machine learning theory with neuroscientific findings. For example, we expect brain-inspired models such as HF-PGM (Taniguchi et al., 2022) to be demonstrated on real robots. The relationship between the free-energy principle (Friston et al., 2012) and spatial cognition in robots is also very interesting. As discussed in several papers on our topic, it would be useful, from an engineering point of view, to construct brain-referenced autonomous intelligence.

## Author contributions

All authors listed have made a substantial, direct, and intellectual contribution to the work and approved it for publication.

## Conflict of interest

Author MS was employed by Sony Computer Science Laboratories. Author HY was employed by The Whole Brain Architecture Initiative. The remaining authors declare that the research was conducted in the absence of any commercial or financial relationships that could be construed as a potential conflict of interest.

## Publisher's note

All claims expressed in this article are solely those of the authors and do not necessarily represent those of their affiliated organizations, or those of the publisher, the editors and the reviewers. Any product that may be evaluated in this article, or claim that may be made by its manufacturer, is not guaranteed or endorsed by the publisher.
